# Epidemiology, Ventilation Management and Outcomes of COPD Patients Receiving Invasive Ventilation for COVID-19—Insights from PRoVENT-COVID

**DOI:** 10.3390/jcm12185783

**Published:** 2023-09-05

**Authors:** Athiwat Tripipitsiriwat, Orawan Suppapueng, David M. P. van Meenen, Frederique Paulus, Markus W. Hollmann, Chaisith Sivakorn, Marcus J. Schultz

**Affiliations:** 1 Division of Respiratory Disease and Tuberculosis, Department of Medicine, Faculty of Medicine Siriraj Hospital, Mahidol University, Bangkok 10400, Thailand; athiwattri@gmail.com; 2 Division of Clinical Epidemiology, Department of Research, Faculty of Medicine Siriraj Hospital, Mahidol University, Bangkok 10400, Thailand; aorstat@gmail.com; 3 Department of Intensive Care, Amsterdam UMC, Location AMC, 1105 AZ Amsterdam, The Netherlands; f.paulus@amsterdamumc.nl (F.P.); marcus.j.schultz@gmail.com (M.J.S.); 4 Department of Anesthesiology, Amsterdam UMC, Location AMC, 1105 AZ Amsterdam, The Netherlands; m.w.hollmann@amsterdamumc.nl; 5 Center of Expertise Urban Vitality, Faculty of Health, Amsterdam University of Applied Sciences, 1101 CD Amsterdam, The Netherlands; 6 Intensive Care Unit, University College London Hospital, London NW1 2BU, UK; chaisith.sivakorn@nhs.net; 7 Mahidol–Oxford Tropical Medicine Research Unit (MORU), Mahidol University, Bangkok 10400, Thailand; 8 Nuffield Department of Medicine, University of Oxford, Oxford OX3 7BN, UK; 9 Department of Anesthesia, General Intensive Care and Pain Management, Division of Cardiothoracic and Vascular Anesthesia & Critical Care Medicine, Medical University of Vienna, 1090 Vienna, Austria

**Keywords:** COPD, ARDS, COVID-19, invasive ventilation, ventilation management, outcome

## Abstract

Chronic obstructive pulmonary disease (COPD) is a risk factor for death in patients admitted to intensive care units (ICUs) for respiratory support. Previous reports suggested higher mortality in COPD patients with COVID-19. It is yet unknown whether patients with COPD were treated differently compared to non-COPD patients. We compared the ventilation management and outcomes of invasive ventilation for COVID-19 in COPD patients versus non-COPD patients. This was a post hoc analysis of a nation-wide, observational study in the Netherlands. COPD patients were compared to non-COPD patients with respect to key ventilation parameters. The secondary endpoints included adjunctive treatments for refractory hypoxemia, and 28-day mortality. Of a total of 1090 patients, 88 (8.1%) were classified as having COPD. The ventilation parameters were not different between COPD patients and non-COPD patients, except for FiO_2_, which was higher in COPD patients. Prone positioning was applied more often in COPD patients. COPD patients had higher 28-day mortality than non-COPD patients. COPD had an independent association with 28-day mortality. In this cohort of patients who received invasive ventilation for COVID-19, only FiO_2_ settings and the use of prone positioning were different between COPD patients and non-COPD patients. COPD patients had higher mortality than non-COPD patients.

## 1. Introduction

Chronic obstructive pulmonary disease (COPD) is a common airway condition that affects around 10% of the world’s population and causes approximately 3,000,000 deaths each year [1]. COPD has been linked to a higher risk of mortality in a variety of respiratory tract infections, including bacterial [2] and viral pneumonia [3]. COPD is also considered a risk factor for death in patients who need admission to an intensive care unit (ICU) for respiratory support [4,5], though mortality in these patients mainly depends on the cause of respiratory failure.

The coronavirus disease 2019 (COVID-19) pandemic unavoidably afflicted this large group of patients. Previous reports suggested a higher mortality rate in COPD patients with COVID-19 [6]. It is yet unknown whether patients with a history of COPD were treated differently compared to non-COPD patients. In particular, the ways in which invasive ventilation was applied might have been different. There may also have been differences in how refractory hypoxemia was treated. Such differences, if any, could have affected patient outcomes.

We conducted a post hoc analysis of a conveniently sized multicenter observational study, named ‘Practice of Ventilation in COVID-19’ (PRoVENT-COVID) [7]. Herein, we determined and compared ventilator settings and ventilation parameters, supportive treatments for refractory hypoxemia and outcomes in COPD patients versus non-COPD patients. We hypothesized that ventilation management in COPD patients would be different from that in non-COPD patients. We also determined which factors had an independent association with outcomes.

## 2. Materials and Methods

### 2.1. Study Design

This is a post hoc analysis of PRoVENT-COVID, a nation-wide, multicenter, observational cohort study [7]. PRoVENT-COVID included patients in 22 ICUs in the Netherlands. The study protocol was approved by the Institutional Review Board of the Amsterdam University Medical Centers, ‘AMC’ location. Members of the PRoVENT-COVID steering committee were responsible for the recruitment of study sites; local investigators and data collectors sought approval from their respective Institutional Review Boards or Research Ethics Committees. The study protocol was prepublished [8], and the study was registered at ClinicalTrials.gov (NCT04346342). The need for individual informed consent was waived due to the observational nature of this investigation. The study coordinators and trained data collectors assisted local doctors and monitored the study according to the International Conference on Harmonization’s Good Clinical Practice Guideline, ensuring the integrity and timely completion of data collection.

### 2.2. Patients

Patients were eligible for participation if: (1) they were aged 18 years or older; (2) they had been admitted to one of the participating ICUs in the first wave of the national outbreak; (3) had acute respiratory failure related to COVID-19; and (4) required invasive ventilation. COVID-19 was confirmed via RT–PCR in all patients. Patients who received noninvasive ventilation, and patients who were transferred to a non-participating ICU within 1 h after intubation and underwent invasive ventilation, were excluded. For the current analysis, we pragmatically excluded patients under the age of 40 years, to improve the accuracy of the history of COPD.

### 2.3. Patient Classification

Patients with a known history of COPD were classified as COPD patients; patients without a known history of COPD were classified as non-COPD patients. History of COPD was based on information recorded in the medical records, which was collected for PRoVENT-COVID.

### 2.4. Collected Data

Demographic data, the severity of illness scores expressed in Acute Physiology and Chronic Health Evaluation (APACHE) scores II or IV, Simplified Acute Physiology Score (SAPS) II or the Sequential Organ Failure Assessment (SOFA) score were collected at baseline. Trained data collectors scored chest imaging performed to determine the extent of lung involvement; chest X-rays were scored as having opacities in one, two, three or four quadrants; chest computed tomography (CT) scans were scored as having 0%, 25%, 50%, 75% or 100% involvement. ARDS severity was categorized using the current Berlin definition of ARDS [9]. Laboratory tests, including arterial blood gas, lactate and serum creatinine, were collected at baseline.

Ventilator settings and parameters were collected after the first hour of invasive ventilation, and thereafter at fixed time points (08:00 a.m., 4:00 p.m. and 12:00 p.m.) over the first four calendar days of ventilation. The first day a patient received invasive ventilation in a participating ICU was named ‘day 0’. Adjunctive treatments of refractory hypoxemia were also recorded during those four days, including the use of recruitment maneuvers, prone positioning and neuromuscular blocking agents. Typical ICU events and complications, including pneumothorax, thromboembolic complications, extubation and re-intubation, tracheostomy and acute kidney injury were collected up to day 28. At day 90, the intubation status, day of discharge from the ICU and hospital, and day of death in non-survivors were recorded.

### 2.5. Calculations

The driving pressure (ΔP) was calculated by subtracting the positive end-expiratory pressure (PEEP) from the plateau pressure (P_plat_) during volume-controlled ventilation, or from the maximum airway pressure (P_max_) during pressure-controlled ventilation, and only at timepoints with evidence of the absence of spontaneous breathing. The dead space fraction was calculated by subtracting the end-tidal carbon dioxide (et–CO_2_) from the arterial carbon dioxide pressure (PaCO_2_) and dividing by PaCO_2_. Respiratory system compliance (C_RS_) was calculated by dividing the tidal volume (V_T_) by ΔP. The mechanical power of ventilation (MP) was calculated from V_T_, respiratory rate (RR), peak pressure (P_peak_) and ΔP (0.098 × 2217V_T_ × RR × [P_peak_ − 0.5 × ΔP]); if P_peak_ was not available, we used P_plat_ (0.098 × V_T_ × RR × [P_plat_ − 0.5 × ΔP]). The number of days free from the ventilator at day 28 (VFD–28) was defined as the number of days a patient was not connected to a ventilator in the first 28 days after the start of ventilation, wherein patients who died before day 28 days received zero free days, even if weaned from ventilation within this timeframe.

### 2.6. Endpoints

The primary endpoint was marked by the collection of key ventilator settings and ventilation parameters over the first four calendar days of invasive ventilation, including V_T_, PEEP, ΔP and C_RS_. The secondary endpoints included other settings and parameters, including the mode of ventilation, alveolar minute ventilation (AMV), P_peak_, RR, fraction of inspired oxygen (FiO_2_), MP, dead space fraction and arterial blood gas analysis results, and et-CO_2_. The other secondary endpoints were the use of adjunctive therapies, typical ICU events and complications, the duration of ventilation, the length of ICU and hospital stays, the number of VFD–28 and 28-day mortality.

### 2.7. Power Calculation

We did not perform a formal power calculation; instead, the sample size was based on the number of patients included in the original study.

### 2.8. Statistical Analysis

Quantitative data are presented as mean ± standard deviation or median with interquartile ranges were appropriate. Categorical data are presented as numbers and proportions. A Chi-square test or Fisher’s exact test were used to compare categorical variables. An independent *t*-test or Mann–Whitney U test was used to compare continuous data. Cumulative distribution plots were created for the ventilator settings and parameters to visualize differences between COPD and non-COPD patients.

To assess the mortality impact of COPD, hazard ratios were calculated using shared frailty adjusted Cox regression with the center set as frailty for mortality. The subdistribution hazard ratios were also calculated for ICU and hospital length of stay, and the duration of ventilation, using a Fine–Gray competing risk analysis with death as the competing risk. Forward stepwise selection was used, defined by *p* < 0.2 according to a univariable analysis of the two groups, which were added to a multivariable model to demonstrate the impact of COPD on 28-day mortality. These included age; sex; body mass index; dead space fraction; PaO_2_/FiO_2_; plasma creatinine; history of hypertension, heart failure, diabetes mellitus, chronic kidney disease and active malignancy; the use of angiotensin-converting enzyme inhibitors; the use of angiotensin II receptor blockers; the use of a vasopressor or inotropes; fluid balance; pH; mean arterial pressure; heart rate; and C_RS_.

All analyses were performed in STATA statistics version 14 (StataCorp, College Station, TX, USA). A *p*-value of <0.05 was considered statistically significant.

## 3. Results

### 3.1. Patients

Between 1 March and 1 June 2020, 1122 patients were included in PRoVENT-COVID. The main reasons for exclusion were not having received invasive ventilation or having an alternative diagnosis for acute hypoxemic respiratory failure. Of the remaining 1090 patients, 88 (8.1%) were classified as COPD patients (Supplement Figure S1). COPD patients were older and used corticosteroids, angiotensin-converting enzyme inhibitors and angiotensin ll receptor blockers more often than non-COPD patients (Table 1). At baseline, COPD patients had lower PaO_2_/FiO_2_, lower et–CO_2_ and a higher dead space fraction (Table 2). ARDS was classified as severe more often in COPD patients, but none of the severity of disease scores were different between the two groups.

### 3.2. Ventilation Management

**Figure 1:**
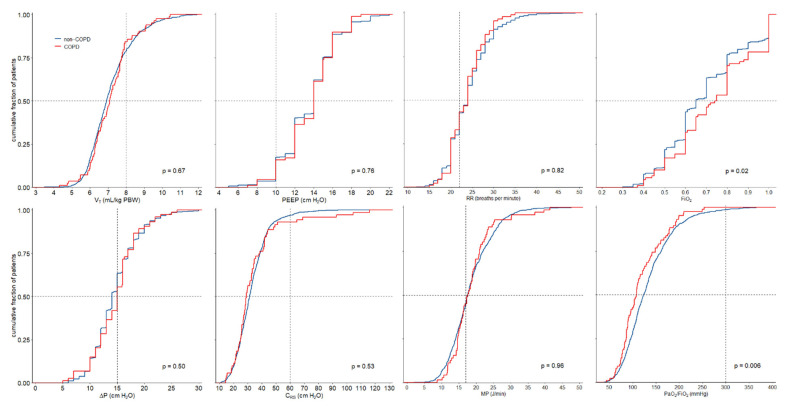
Cumulative distribution of ventilatory characteristics. The worst available value for each parameter was used.

Ventilation management is detailed in Table 2 and Figure 1. V_T_ and PEEP were not different between COPD and non-COPD patients. There were also no differences between ΔP and C_RS_. COPD patients were ventilated with higher FiO_2_. COPD patients also had lower arterial pH, lower etCO_2_ and higher dead space fractions.

Of the adjunctive treatments for refractory hypoxemia, prone positioning was used more often in COPD patients (Supplement Table S1). There were no differences in the use of recruitment maneuvers or neuromuscular blocking agents.

### 3.3. Outcomes

**Figure 2:**
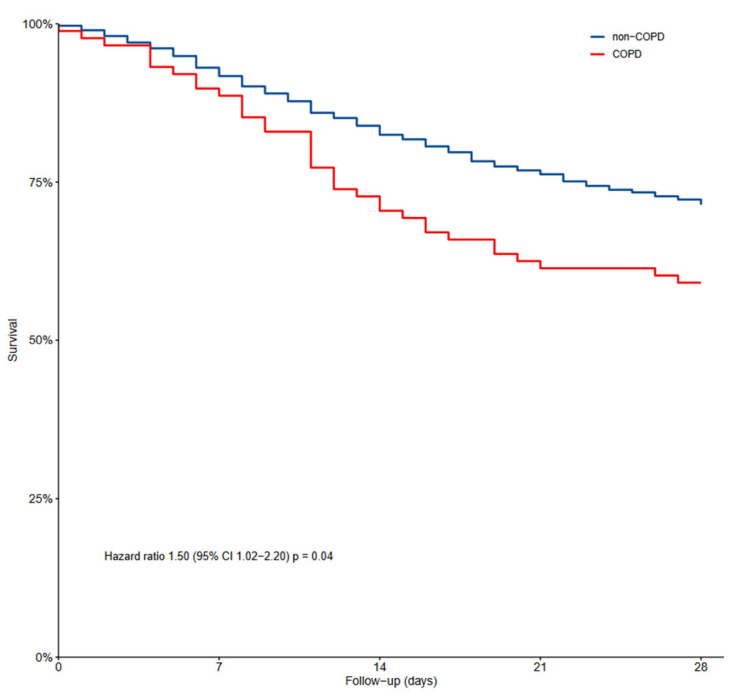
Kaplan–Meier graph showing mortality in COPD patients and non-COPD patients.

Air leaks, thromboembolic complications, acute kidney injury and re-intubations occurred as often in COPD patients as in non-COPD patients (Table 3). The duration of ventilation and the number of VFD–28 patients were not different between COPD and non-COPD patients (Figure 2). COPD patients had higher ICU and in-hospital mortality, and also higher 28-day and 90-day mortality.

In the multivariable analysis, COPD was an independent risk factor for 28-day mortality (Supplement Table S2). Fine–Gray competing risk analysis with death as the competing risk showed that the duration of ventilation, ICU length of stay and hospital length of stay were longer in COPD patients compared to non-COPD patients (Supplement Figure S2).

## 4. Discussion

The main findings of this post hoc analysis of a nation-wide, multicenter, observational study of invasively ventilated COVID-19 patients can be summarized as follows: (1) compared to non-COPD patients, COPD patients had more severe hypoxemia and ARDS; (2) the key ventilator settings and parameters were not different between COPD and non-COPD patients; (3) COPD patients were ventilated with higher FiO_2_ and had lower PaO_2_/FiO_2_; (4) COPD patients had lower arterial pH and et–CO_2_, and a higher dead space fraction; and (5) COPD patients received prone positioning more often. In addition, (6) COPD patients had higher mortality than non-COPD patients, and (7) COPD and a history of hypertension were independent risk factors for 28-day mortality.

Our study has several strengths. This analysis is one of the first to investigate ventilation management in COPD patients who received invasive ventilation for COVID-19. Trained investigators collected granular ventilation data over the first four days, increasing the robustness of the data. Patients were recruited in different types of hospitals, increasing the generalizability of our findings. The caregivers were not aware of the study at the time of data collection, minimizing the risk of observation bias. We had a sophisticated pre-defined statistical analysis plan in place, which was strictly followed.

The findings of this study extend our knowledge of ventilation practices in COPD patients with COVID-19. To the best of our knowledge, this study is the first to compare ventilation management between COPD and non-COPD patients in the context of COVID-19 in such great detail. The similarity in ventilator practices may not be unexpected given that both groups suffered from severe acute hypoxemic respiratory failure. The main difference between the groups was the severity of gas exchange abnormalities, resulting in the use of higher FiO_2_ and more frequent use of prone positioning for refractory hypoxemia.

The best practice in invasive ventilation in COPD patients with ARDS remains uncertain. It is questionable whether low V_T_ ventilation should be used in COPD patients as strictly as has been advised for ARDS patients [10]. It is also uncertain whether PEEP titration should follow PEEP/FiO_2_ tables as in ARDS patients [11], especially because COPD patients may be at increased risk of dynamic overinflation with deleterious consequences [11,12]. The findings of a previous study using electrical impedance tomography to determine the best PEEP in ARDS patients suggested that PEEP in COPD patients should be lower than that based on a PEEP/FiO_2_ table [13]. In a study of adaptive support ventilation, PEEP was also lower in COPD patients than in patients with ARDS, but this study did not include patients with COPD with ARDS [14]. Notably, Practice of Ventilation was similar between COPD patients and non-COPD patients. There are several possible explanations for this finding. First, it is quite possible that during the firsts months of the pandemic, caregivers were not sure how to ventilate COVID-19 patients, let alone COVID-19 patients with COPD. It could also be that because of the severity of gas exchange impairment, it was not possible to apply different strategies. Lastly, it could be that patients with severe or exacerbated COPD were not admitted to the ICU during this time as there was a shortage of ICU beds, leading to COPD patients admitted to ICU being ventilated in a similar way to non-COPD patients.

We found a higher dead space fraction in COPD patients compared to non-COPD patients. This is, at least in part, in line with previous studies that showed a higher dead space fraction in ventilated COPD patients for reasons other than COVID-19 [15]. The higher dead space fraction in COPD patients in our cohort may, at least to some extent, be due to the application of a too high a level of PEEP [16,17]. However, there are no clinical trials that compare the effects of different levels of PEEP, either on the dead space fraction or on outcomes, in COPD patients with ARDS. Similarly, there are no clinical trials of prone positioning in this patient group, and such studies remain needed to determine the best ventilation practice in COPD patients with ARDS.

COPD is a risk factor for mortality in critically ill invasively ventilated patients [4,18]. Our findings extend this knowledge by showing that COPD is a risk factor for death in critically ill invasively ventilated COVID-19 patients, independent of age, sex, BMI, PaO_2_/FiO_2_, comorbidities and the use of antihypertensive drugs. COPD was also associated with a prolonged length of stay in the ICU and in hospitals as well as a prolonged duration of ventilation. Notably, the use of lower PEEP is suggested in patients with COPD [13]. In this study, COPD patients received a level of PEEP comparable to non-COPD patients, possibly adding to worse outcomes in the COPD patients.

The main limitation of our study is that presence of COPD was based on whether this was reported in the medical record. It could have been that clinicians also scored COPD in cases of asthma and other chronic airway diseases, thereby over-diagnosing COPD, or that patients with undiagnosed COPD were scored as not having COPD, or that COPD diagnosis was influenced mainly by smoking history, leading to under-reporting. This study also did not allow us to capture spirometry data. For these reasons, we restricted our analysis to patients aged older than 40 years [19]. Furthermore, data on the use of bronchodilating drugs were not collected, and whether or not patients received these drugs could have influenced their outcomes. We restricted the collection of data on ventilation characteristics and adjunctive therapy to the first four days of invasive ventilation. After these days, ventilation may have been different between the two patient groups, and we cannot exclude the possibility that ventilator management after the first four days of ventilation affects outcomes. Finally, as this is an analysis of an observational study, no causality can be claimed and the results should be seen as exploratory.

## 5. Conclusions

In this cohort of critically ill patients who received invasive ventilation for acute hypoxemic respiratory failure due to COVID-19, ventilation management was not different between COPD and non-COPD patients, except for FiO_2_ settings and the use of prone positioning. COPD had independent associations with 28-day mortality.

## Figures and Tables

**Table 1:** Patient demographics and baseline characteristics.

	COPD Patients N = 88	Non-COPD Patients N = 1002	*p*-Value
**Demographics**			
Age, year, median [IQR]	68 [62–72]	65 [58–72]	0.03
Male sex, n (%)	60 (68.2)	735 (73.4)	0.30
BMI, kg/m^2^, median [IQR]	28 [26–30]	28 [25–31]	0.84
**Severity of illness**			
SAPS II, median [IQR]	35 [30–48]	36 [29–43]	0.32
APACHE II score, median [IQR]	15 [13–20]	16 [12–20]	0.41
APACHE IV score, median [IQR]	55 [49–71]	56 [45–69]	0.33
SOFA score, median [IQR]	8 [6–11]	7 [6–10]	0.73
**Severity of ARDS, n (%)**			0.01
No ARDS	2 (2.4)	16 (1.6)	
Mild	1 (1.2)	104 (10.7)	
Moderate	50 (59.5)	602 (61.7)	
Severe	31 (36.9)	254 (26.0)	
**Co-existing disorders, n (%)**			
Hypertension	28 (31.8)	351 (35.0)	0.54
Heart failure	3 (3.4)	45 (4.5)	1.00
Diabetes mellitus	13 (14.8)	236 (23.6)	0.06
Chronic kidney disease	5 (5.7)	41 (4.1)	0.41
Liver cirrhosis	0 (0.0)	3 (0.3)	1.00
Active hematological neoplasia	0 (0.0)	16 (1.6)	0.63
Active solid neoplasia	4 (4.5)	23 (2.3)	0.27
Neuromuscular disease	0 (0.0)	7 (0.7)	1.00
Immunosuppression	2 (2.3)	22 (2.2)	1.00
**Current medication, n (%)**			
Systemic steroids	7 (8.0)	31 (3.1)	0.03
Inhaled steroids	48 (54.5)	73 (7.3)	<0.001
Angiotensin-converting enzyme inhibitors	8 (9.1)	181 (18.1)	0.03
Angiotensin II receptor blockers	16 (18.2)	111 (11.1)	0.05
Beta-blockers	21 (23.9)	189 (18.9)	0.25
Insulin	3 (3.4)	75 (7.5)	0.16
Metformin	8 (9.1)	166 (16.6)	0.07
Statins	30 (34.1)	300 (29.9)	0.42
Calcium channel blockers	19 (21.6)	176 (17.6)	0.35
**Chest imaging**			
Chest CT scan performed, n (%)	25 (29.4)	321 (33.6)	0.43
Lung parenchyma affected at chest CT, n (%)			0.70
<25%	11 (44.0)	115 (35.8)	
50%	7 (28.0)	92 (28.7)	
75%	6 (24.0)	95 (29.6)	
100%	1 (4.0)	19 (5.9)	
Lung parenchyma affected at CXR, number of quadrants, n (%)			0.48
1	4 (8.0)	37 (7.0)	
2	14 (28.0)	118 (22.2)	
3	16 (32.0)	146 (27.5)	
4	16 (32.0)	230 (43.3)	
**Laboratory tests**			
Plasma lactate, mmol/L, median [IQR]	1.2 [0.9–1.4]	1.2 [0.9–1.5]	0.44
Plasma creatinine, µmol/L (median [IQR])	77 [60–101]	78 [63–98]	0.89

Abbreviations: BMI, body mass index; COPD, chronic obstructive pulmonary disease; APACHE, Acute Physiology and Chronic Health Evaluation; SOFA, Sequential Organ Failure Assessment; ARDS, acute respiratory distress syndrome; CT, computed tomography; CXR, chest X-ray.

**Table 2:** Mechanical ventilation use during the first day of mechanical ventilation.

	COPD Patients N = 88	Non-COPD Patients N = 1002	*p*-Value
**Mode of mechanical ventilation, n (%)**			0.12
Volume-controlled	18 (21)	143 (14)	
Pressure-controlled	41 (47)	561 (56)	
Pressure support	3 (3)	50 (5)	
SIMV	9 (10)	72 (7)	
APRV	5 (6)	27 (3)	
INTELLiVENT–ASV	5 (6)	36 (4)	
Other	6 (7)	109 (11)	
**Ventilation Parameters**			
Expiratory V_T_, mL, median [IQR]	440 [387–498]	451 [408–502]	0.13
V_T_ per PBW, mL/kg, median [IQR]	6.2 [5.9–7.0]	6.4 [5.9–7.0]	0.67
PEEP, cmH_2_O, median [IQR]	13 [12–15]	13 [11–15]	0.24
Total Respiratory rate, median [IQR]	22 [20–24]	22 [19–24]	0.84
FiO_2_, median [IQR]	0.6 [0.5–0.7]	0.6 [0.5–0.7]	0.01
P_peak_, cmH_2_O, median [IQR]	27 [24–29]	27 [24–30]	0.78
Driving pressure, cmH_2_O, median [IQR]	14 [12–16]	14 [12–16]	0.87
Compliance, cmH_2_O/L, median [IQR]	32 [26.8–39]	33 [27–40]	0.70
Mechanical power, J/min, median [IQR]	18 [15–20]	19 [16–22]	0.07
Minute ventilation, L/min, median [IQR]	9 [8–10]	10 [8–11]	0.07
pH, median [IQR]	7.35 [7.29–7.39]	7.37 [7.31–7.41]	0.02
PaO_2_, kPa, median [IQR]	10 [9–12]	11 [9–13]	0.08
PaO_2_/FiO_2_, mmHg, median [IQR]	114 [89–149]	128 [99–168]	0.01
PaCO_2_, kPa, median [IQR]	6.1 [5.5–6.5]	5.9 [5.2–6.7]	0.25
End-tidal CO_2_, kPa, median [IQR]	4.6 [4.1–5.3]	4.9 [4.4–5.6]	0.01
Dead space fraction, median [IQR]	0.24 [0.14–0.33]	0.16 [0.06–0.26]	<0.001

Abbreviations: APRV, airway pressure release ventilation; ASV, adaptive support ventilation; COPD, chronic obstructive pulmonary disease; FiO_2_, fraction of inspired oxygen; IQR; interquartile range; J/min, joules per minute; kg, kilogram; kPa, kiloPascal; mL, milliliter; PaCO_2_, arterial pressure of carbon dioxide; PaO_2_, arterial pressure of oxygen; PBW, predicted body weight; PEEP, positive end-expiratory pressure; P_peak_, peak airway pressure; SIMV, synchronized intermittent mandatory ventilation; V_T_, tidal volume.

**Table 3:** Clinical outcomes and ICU complications.

	All N = 1090	COPD N = 88	Non-COPD N = 1002	*p*-Value
28-day mortality, n (%)	319 (29%)	36 (41%)	283 (28%)	0.02
90-day mortality, n (%)	369 (34%)	39 (44%)	330 (33%)	0.04
In-hospital mortality, n (%)	364 (37%)	39 (49%)	325 (36%)	0.02
ICU mortality, n (%)	354 (33%)	38 (45%)	316 (32%)	0.02
Length of hospital stay, days, median [IQR]	23 [14–37]	20 [11–31]	24 [14–37]	0.06
Length of ICU stay, days, median [IQR]	15 [9–26]	12 [8–24]	16 [9–26]	0.11
Ventilator-free days at day 28, days, median [IQR]	16 [10–28]	14 [10–30]	16 [10–28]	0.92
Duration of ventilation, days, median [IQR]	14 [8–23]	11 [8–20]	14 [8–23]	0.07
Tracheostomy, n (%)	187 (17%)	14 (16%)	173 (17%)	0.76
Pneumothorax, n (%)	41 (4%)	4 (5%)	37 (4%)	0.57
**Thromboembolic complications, n (%)**				
Pulmonary embolism	244 (22%)	20 (23%)	224 (22%)	0.94
Deep vein thrombosis	53 (5%)	5 (6%)	48 (5%)	0.61
Ischemic stroke	31 (3%)	3 (3%)	28 (3%)	0.73
Myocardial infarction	16 (1%)	0 (0%)	16 (2%)	0.63
Systemic arterial thrombosis	4 (0%)	1 (1%)	3 (0%)	0.29
Acute kidney injury, n (%)	488 (45%)	38 (43%)	450 (45%)	0.73
Re-intubation, n (%)	138 (13%)	8 (9%)	130 (13%)	0.30

Abbreviations: COPD, chronic obstructive pulmonary disease; ICU, intensive care unit; IQR, interquartile range.

## Data Availability

The data used in this study are available upon request to the steering committee of the PRoVENT-COVID study.

## Supplementary Materials

The following supporting information can be downloaded at: https://www.mdpi.com/article/10.3390/jcm12185783/s1, Supplementary Table S1. Rescue therapies for refractory hypoxemia, and other therapies during the first four days of mechanical ventilation; Supplementary Table S2. Multivariable analysis of factors in 28-day mortality; Supplementary Figure S1. CONSORT diagram; Supplementary Figure S2. Competing risk analysis for ICU length of stay, hospital length of stay and duration of ventilation with death as the competing risk.

## Registration

PRoVENT-COVID is registered at ClinicalTrials.gov (NCT04346342).

## Appendix A

**PRoVENT-COVID Investigators:** PRoVENT-COVID stands for the Practice of Ventilation in COVID-19 Patients study. Investigator list in alphabetical order: S. Ahuja; J.P. van Akkeren; A.G. Algera; C.K. Algoe; R.B. van Amstel; P. van de Berg; D.C. Bergmans; D.I. van den Bersselaar; F.A. Bertens; A.J. Bindels; J.S. Breel; C.L. Bruna; M.M. de Boer; S. den Boer; L.S. Boers; M. Bogerd; L.D. Bos; M. Botta; O.L. Baur; H. de Bruin; L.A. Buiteman–Kruizinga; O. Cremer; R.M. Determann; W. Dieperink; J. v. Dijk; D.A. Dongelmans; M.J. de Graaff; M.S. Galekaldridge; L.A. Hagens; J.J. Haringman; S.T. van der Heide; P.L. van der Heiden; L.L. Hoeijmakers; L. Hol; M. W. Hollmann; J. Horn; R. van der Horst; E.L. Ie; D. Ivanov; N.P. Juffermans; E. Kho; E.S. de Klerk; A.W. Koopman; M. Koopmans; S. Kucukcelebi; M.A. Kuiper; D.W. de Lange; I. Martin–Loeches; G. Mazzinari; D.M. van Meenen; N. van Mourik; S.G. Nijbroek; E.A. Oostdijk; F. Paulus; C. J. Pennartz; J. Pillay; I.M. Purmer; T.C. Rettig; O. Roca; J.P. Roozeman; M.J. Schultz; A. Serpa Neto; G.S. Shrestha; M.E. Sleeswijk; P.E. Spronk; A.C. Strang; W. Stilma; P. Swart; A.M. Tsonas; C.M.A. Valk; A.P. Vlaar; L.I. Veldhuis; W.H. van der Ven; P. van Velzen; P. van Vliet; P. van der Voort; L. van Welie; B. van Wijk; T. Winters; W.Y. Wong; A.R. van Zanten.
