# Supplementary material for: Epidemiology, Ventilation Management and Outcomes of COPD Patients Receiving Invasive Ventilation for COVID-19—Insights from PRoVENT-COVID
Source: J Clin Med. 2023 Sep 5;12(18):5783. doi: 10.3390/jcm12185783 (PMC10532133; doi:10.3390/jcm12185783)
Supplement: Supplementary file 1 [file jcm-12-05783-s001.zip › jcm-2544124-supplementary.pdf]

Online supplement to

Epidemiology, Ventilation Management and Outcome of COPD Patients Receiving Invasive Ventilation for COVID–19—insights from PRoVENT–COVID

Athiwat Tripipitsiriwat [1], Orawan Suppapueng [2], David M. P. van Meenen [3,4,*], Frederique Paulus [3,5], Markus W. Hollmann [4], Chaisith Sivakorn [6] and Marcus J. Schultz [3,7,8,9,†] on behalf of the PRoVENT-COVID Investigators

Supplement Table S1. Rescue therapies for refractory hypoxemia, and other therapies during the first four days of mechanical ventilation.

	**COPD patients N = 88**	**non–COPD patients N = 1002**	**P–value**
Use of recruitment maneuvers, n (%)	4 (5.8)	59 (7.0)	1.00
Number of days with prone positioning, days, median [IQR]	2 [0–4]	1 [0–3]	0.01
Number of days with NMBA use, days, median [IQR]	0 [0–2]	0 [0–2]	0.78
Use of ECMO within first 4 days, n (%)	0 (0.0)	11 (1.1)	1.00
Number of days with vasopressors or inotropes, days, median [IQR]	3 [2–4]	3 [2–4]	0.97
Use of RRT within first 4 days, n (%)	26 (29.5)	260 (25.9)	0.46

Abbreviations: COPD, chronic obstructive pulmonary disease; NMBA, neuromuscular blocking agent; ECMO, extra–corporeal membrane oxygenation; RRT, renal replacement therapy.

Supplement Table S2. Multivariable analysis of factors in 28–day mortality.

	crude HR (95%–CI)	P–value	adjusted HR (95%–CI)	P–value
COPD	1.60 (1.13–2.28)	0.01	1.50 (1.02–2.20)	0.04
**Demographic characteristics**				
Age	1.07 (1.06–1.09)	<0.001	1.01 (1.00–1.03)	0.10
Male sex	1.12 (0.87–1.46)	0.38		
BMI	0.99 (0.97–1.01)	0.32		
**Medical history**				
Hypertension	1.48 (1.18–1.86)	<0.001	1.52 (1.16–2.00)	0.003
Heart failure	1.78 (1.14–2.78)	0.01	1.30 (0.76–2.21)	0.34
Diabetes	1.60 (1.25–2.05)	<0.001	0.88 (0.66–1.15)	0.34
Chronic kidney disease	1.27 (0.77–2.11)	0.35		
Active cancer	1.85 (0.89–3.06)	0.39		
**Home medication**				
Angiotensin converting enzyme inhibitor	1.30 (0.98–1.71)	0.07	1.30 (0.93–1.80)	0.12
Angiotensin II receptor blocker	1.34 (0.97–1.84)	0.08	1.30 (0.89–1.90)	0.17
**Physical factors**				
Plasma creatinine	1.07 (1.00–1.14)	0.04	0.96 (0.83–1.11)	0.58
pH	0.94 (0.91–0.97)	<0.001	0.95 (0.85–1.06)	0.36
PaO_2/FiO_2	1.00 (0.99–1.01)	0.88		
Fluid balance	1.00 (1.00–1.00)	0.03	1.00 (1.00–1.00)	0.18
Use of vasopressor or inotropic	1.13 (0.61–2.10)	0.70	1.27 (0.66–2.45)	0.47
Blood pressure	0.99 (0.98–1.00)	0.21		
Heart rate	1.00 (1.00–1.01)	0.15	1.00 (1.00–1.01)	0.18
**Pulmonary factors**				
Respiratory system compliance	1.00 (0.99–1.01)	0.96		
Dead space fraction	1.01 (0.89–1.14)	0.89		

Abbreviations: HR, hazard ratio; COPD, chronic obstructive pulmonary diseases; BMI, body mass index.

Supplement Figure S1. CONSORT diagram.

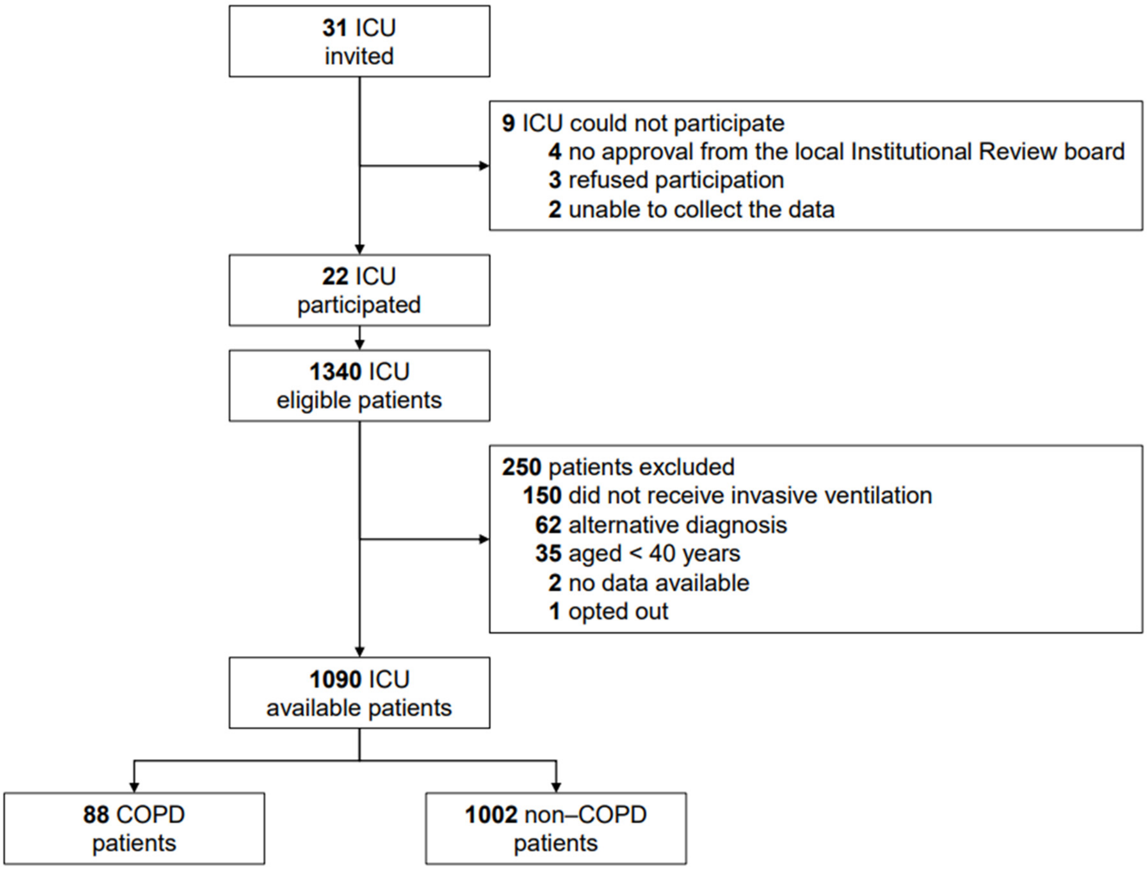

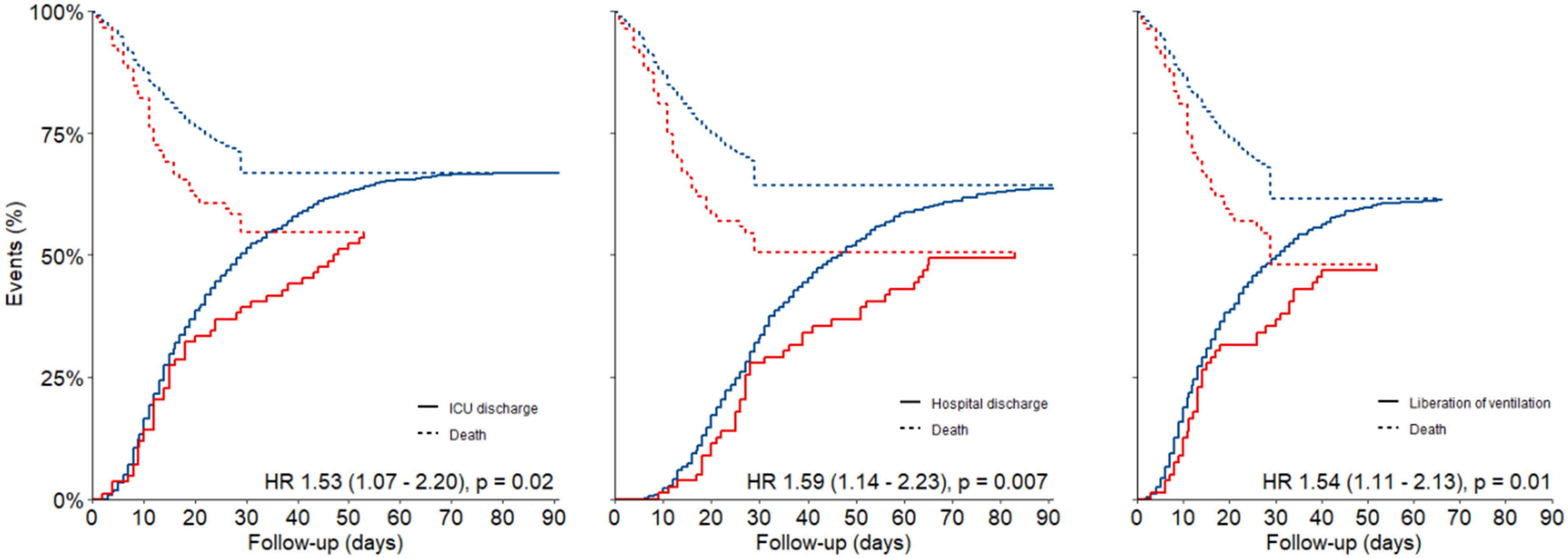

Supplement Figure S2. Competing risk analysis for ICU length of stay, hospital length of stay and duration of ventilation with death as the competing risk.
